# Impact of the COVID-19 pandemic on population-based cancer screening, a nationwide retrospective study in Taiwan

**DOI:** 10.1186/s12913-023-09901-x

**Published:** 2023-08-21

**Authors:** Chih-Hsuan Su, Pi-Shan Hsu, Chu-Sheng Lin

**Affiliations:** 1 Department of Family Medicine, Da-Chien Hospital, Miaoli, 36052 Taiwan; 2 Department of Environment and Occupational Medicine, Taichung Veterans General Hospital, Taichung, 407219 Taiwan; 3 Department of Family Medicine, Taichung Veterans General Hospital, 407219 Taichung, Taiwan; 4 Graduate Institute of Microbiology and Public Health, College of Veterinary Medicine, National Chung-Hsing University, Taichung, Taiwan; 5 Department of Post-Baccalaureate Medicine, College of Medicine, National Chung Hsing University, Taichung, Taiwan; 6 Center for Geriatrics & Gerontology, Taichung Veterans General Hospital, Taichung, 407219 Taiwan

**Keywords:** Oral cancer screening, Breast cancer screening, Colon cancer screening, Cervical cancer screening, Taiwan

## Abstract

**Background:**

The Coronavirus disease 2019 (COVID-19) pandemic has placed a great burden on Taiwan’s health care system. It has also had a great impact on other public health issues, including cancer screening. Delayed cancer screening was also noticed in the U.S. during the pandemic, which may have led to both delayed diagnosis and poor prognosis. In Taiwan, population-based cancer screening for breast cancer, oral cancer, colon cancer and cervical cancer has been executed and ongoing for years.

**Method:**

In this study we have analyzed the change in screening numbers for cancer during the period of the 2019 to 2021 COVID-19 outbreak in Taiwan.

**Results:**

Through our results we found that total cancer screening numbers decreased from 307,547 to 103,289 (a 66% decrease) from the years 2020 to 2021. Specifically, a 63%, 79%, 65% and 71% decrease in screening cases was seen for colon cancer, oral cancer, cervical cancer and breast cancer, respectively, during that period. A similar condition was noticed when comparing 2019 to 2021 when the disclosed total cancer screening numbers decreased by 70% (2019–2021); 65%, 83%, 70% and 76% in colon cancer, oral cancer, cervical cancer and breast cancer, respectively. Among these various cancer screenings, oral cancer screening showed the greatest reduction rate (a drop of 83% compared to 2019 and 79% compared to 2020). We also compared the reduction rates taken from different regions in Taiwan. It was in Taipei, where most COVID-19 cases were noted, that the greatest reduction rate of cancer screening numbers occurred (a drop of76% compared to 2019 and 74% compared to 2020). A proportional decrease of screening cases was also noticed in all areas when confirmed COVID-19 cases rose.

**Conclusions:**

Screening for cancers dropped significantly due to the pandemic and its effect on long-term health needs to be evaluated. Additionally, efforts should be taken to address these cancer screening number deficits which have taken place during the COVID-19 pandemic.

## Background

The Coronavirus disease 2019 (COVID-19) pandemic has had a great impact on health care systems worldwide. Over 428 million people have become infected with the virus, with nearly 6 million people dying due to COVID-19 [1]. Coronavirus initially causes an upper respiratory infection with various symptoms, from common cold symptoms to respiratory failure. While the novel coronavirus epidemic originated from Wuhan City in China, it soon spread worldwide causing a pandemic. Transmission including contact transmissions and aerosol related airborne transmissions were reported [2].

In Taiwan, the first COVID-19 case was discovered on January 21, 2020. Due to Taiwan’s previous experience involving Severe Acute Respiratory Syndrome (SARS), which was also caused by a coronavirus, a rapid and strict response was taken, resulting in a decrease in social events and activities being noticed. Health services were also affected as many Taiwanese hesitated to visit medical institutions, with that same trend being noticed worldwide [3–5].

Cancer is one of the leading causes of death in the United States [6], with screening for it acting as a powerful tool to reduce mortality in some types of cancer. The United States Preventive Services Task Force (USPSTF) recommends people undergo certain types of cancer screening, including breast cancer, colorectal cancer, lung cancer and cervical cancer screening [7–10]. In Taiwan, population-based biennial mammography screening for women aged 45–69 [11], biennial fecal occult blood testing for people aged 50–69 and annual Pap smear screening for women aged 30 years or older [12] have all been conducted for years. Oral cancer screening was launched in 2004, with oral mucosal examinations being performed every two years for people who had the habits of smoking or betel nut chewing, were aged 30 years and older or were aboriginals aged 18 years and older. Effective analyses were conducted which revealed a great reduction in breast cancer mortality [11], as well as the cost effectiveness of both oral cancer screening [13] and cervical cancer screening [14].

There has been previous research performed which has focused on the occurrence of delayed cancer screening due to the COVID-19 pandemic. A sharp decline in the screening rate of breast cancer, colorectal cancer and prostate cancer patients was noticed in 2020 due to the outbreak of the COVID19 pandemic in the USA [15]. Additionally, the COVID-19 pandemic has also had a great impact on referrals after abnormal results were seen upon screening. An increase in avoidable cancer deaths due to delayed diagnosis has also been noticed [16]. In Taiwan, the arrival of COVID-19 was expected due to its geographical location being near China. Here, the first Covid-19 case was confirmed on January 21, 2020, with the number of recorded cases rising sharply in mid-March of the same year due to massive crowds of travelers having returned to Taiwan from endemic regions over the Chinese New Year holiday. Subsequently, a decrease in fecal occult blood test screening rates [17] and mammography screening rates [18, 19] were found. The virus then subsided prior to another outbreak in mid-May 2021 emerging which was much more severe, as more infected people were diagnosed as compared with 2020. Most of the infected people at that time lived in the northern region of Taiwan. While a previous study focused on breast cancer screening and fecal occult blood screening tests, the impact of COVID-19 on both oral cancer screening and cervical cancer screening, which are unique in Taiwan, was not addressed. Additionally, epidemic alert levels were set to Level 3, with actions such as border controls, case identification, quarantining suspicious cases, educating the public, shutting down entertainment venues, forbidding assemblies and banquets, mandatory mask wearing and allocation of medical resources all being executed rapidly at that time. The impact of frequent COVID-19 outbreaks and public health policy surrounding different epidemic alert levels was not however made clear. In our hypothesis, the COVID-19 pandemic may have had a great effect on cancer screening and an inverse relation with distances to/from high COVID-19 prevalent regions. Finally, the COVID-19 pandemic may still be having a different impact on four kinds of cancer screening. The aim of this study is to investigate the impact this outbreak has had and the strict health policies implemented in May 2021 here in Taiwan, as well as their influence on colon, oral, cervical and colon cancer screening.

## Methods

### Ethics statement

This study protocol was approved by the Institutional Review Board of Taichung Veterans General Hospital (IRB no: CE19332B-2). Our study was performed in accordance with the Declaration of Helsinki. The requirement for informed consent was waived by the Institutional Review Board of Taichung Veterans General Hospital due to the retrospective nature of the study.

### Patients

Confirmed COVID-19 case numbers in Taiwan were obtained from the public database of the National Health Command Center. People who were COVID-19 infected as reported by all medical institutions from the years 2019 to 2021 were included for analysis. The number of screenings was acquired from the database of the Cancer Screening and Tracing Information Integrated System of the Health Promotion Administration, Ministry of Health and Welfare. People who underwent cancer screenings including colon, oral, cervical and colon cancer, through the cancer screening program held by the Ministry of Health and Welfare in Taiwan from 2019 to 2021 were included. Data from May 19^th^, 2021 to July 26^th^, 2021, when a Level 3 alert was implemented, was analyzed. A health ID card for confirmation of one’s COVID-19 infectious status and afebrile status needed to be confirmed before entering hospital during this period.

### Study design

We compared the change in screening numbers from the period 2019 to 2021. Screening cases were stratified by both screening items and regions. Screening items involved breast cancer, oral cancer, colon cancer and cervical cancer screening. Regions were divided into 6 groups according to policy of the Health Promotion Administrative and included the Taipei Divisions, Northern Divisions, Central Divisions, Southern Divisions, Kao-Ping Divisions and Eastern Divisions. Most cases of COVID-19 were found in the Taipei Divisions. Thus, we simply analyzed the differences between the Taipei Divisions and the other 5 areas listed above.

### Statistical analysis

**Fig. 1:**
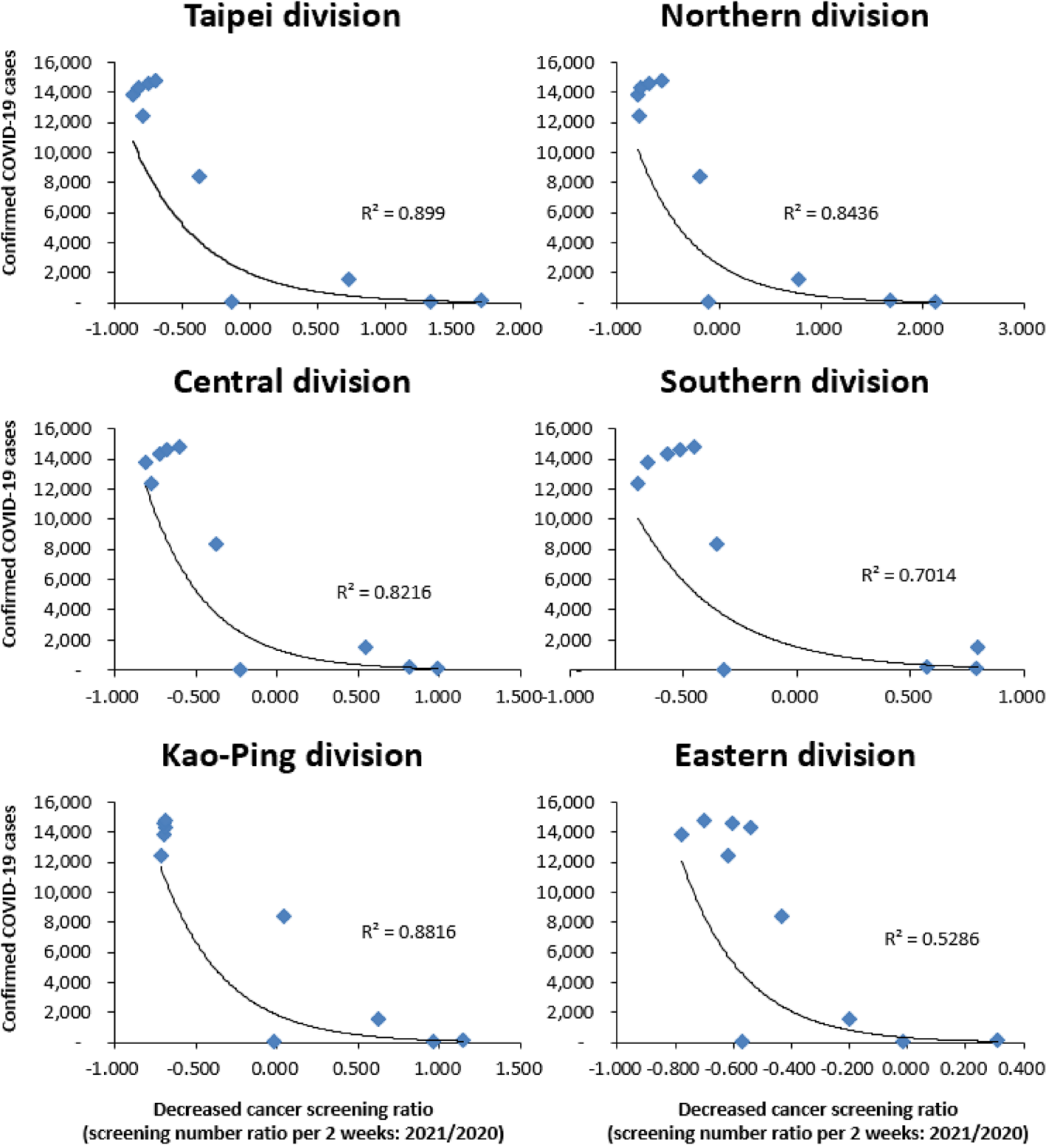
Relation between COVID-19 cumulative cases and difference in proportions during 2021 to 2020

**Fig. 2:**
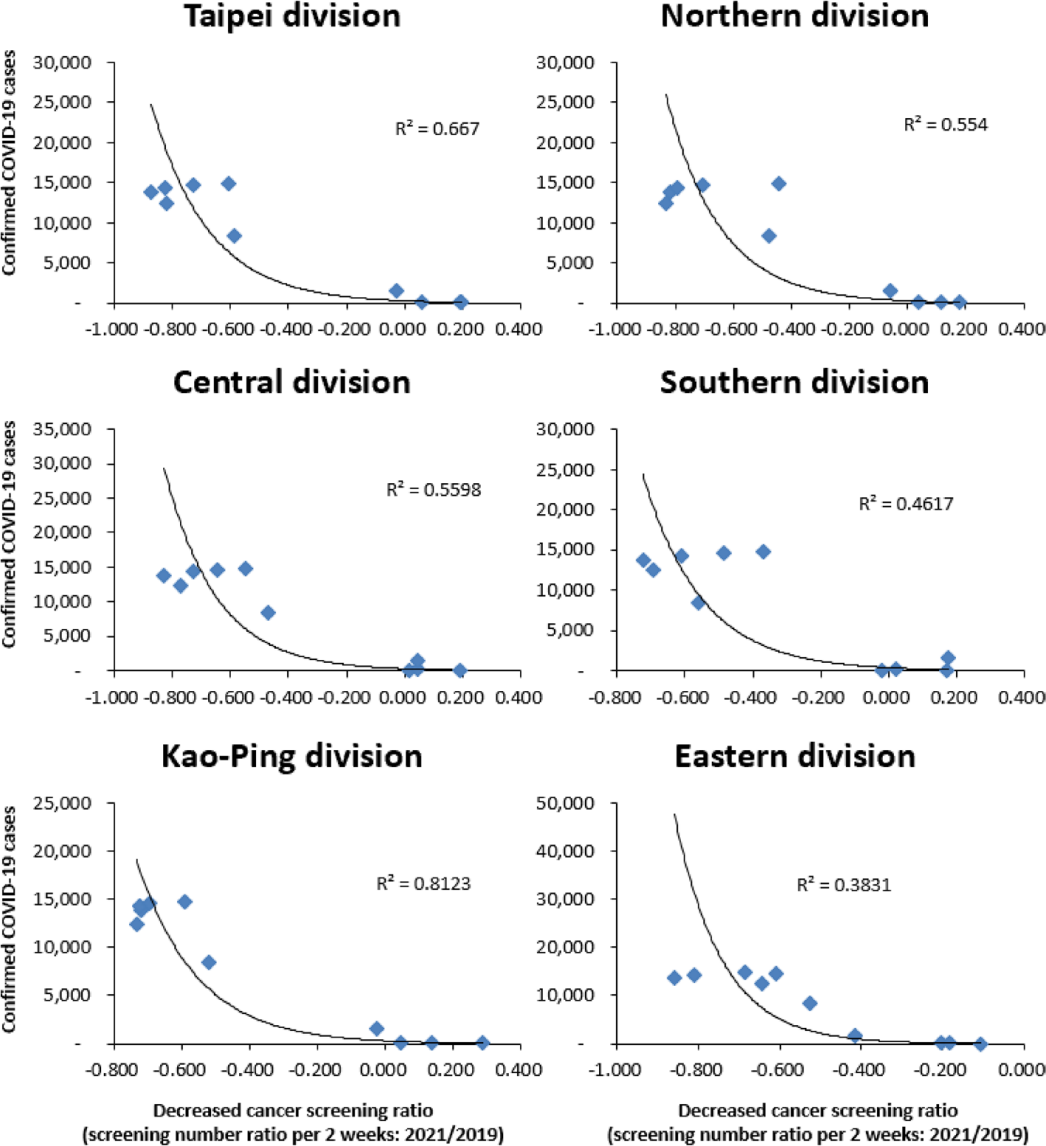
Relation between COVID-19 cumulative cases and difference in proportions during 2021 to 2019

Differences in screening numbers were calculated during the months of May to June over the years 2019–2021 and 2020–2021, and shown in percentages. Exponential Trendline, one type of non-linear regression model, was performed to examine the relation between cumulative outbreak cases and cancer screening numbers. The x-axis in Fig. 1 is the difference between screening number of 2021 and 2020 divided by screening number of 2020. ((screening number of 2021- screening number of 2020)/screening number of 2020) The y-axis represents cumulative confirmed COVID-19 cases. The X-axis in Fig. 2 is the difference between screening number of 2021 and 2019 divided by screening number of 2019. ((screening number of 2021- screening number of 2019)/screening number of 2019) The y-axis represents cumulative confirmed COVID-19 cases. R-square represents how well the trendline fit the data. All statistical analyses were performed using IBM SPSS version 22 (SPSS Inc., Chicago, IL). For all tests, a P value (two-tailed) less than 0.05 was considered statistically significant.

## Results

**Fig. 3:**
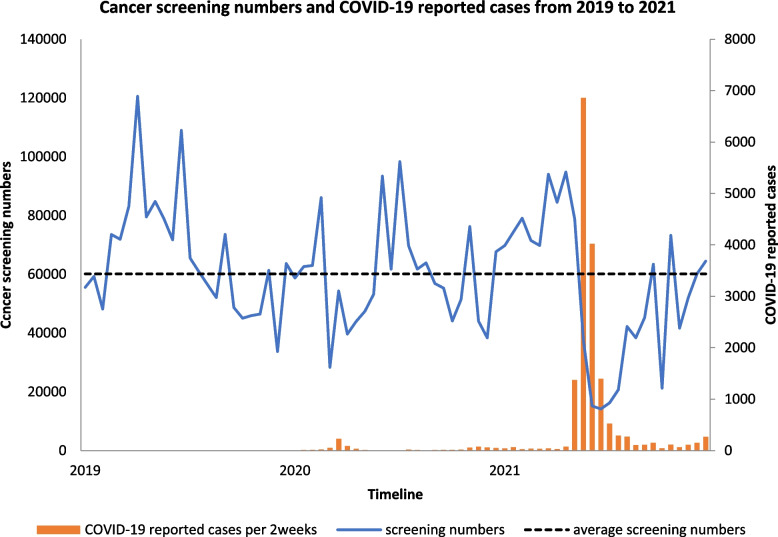
COVID-19 infectious reported from 2019 to 2021 and total screening cases

People who were reported to be COVID-19 infected during the period 2019 to 2021 along with the total screening cases are displayed in Fig. 3. Cancer screening numbers dropped dramatically after the COVID-19 outbreak in May 2021, but rebounded soon after COVID-19 confirmed cases had dropped gradually. As cumulative COVID-19 cases increased, decreased cancer screen ratio was noticed in Figs. 1 and 2, no matter compared to 2020 or 2019. There were 103,829, 307,547 and 348,732 screening cases during the period May to July in 2021, 2020, 2019, respectively (Table 1). The cancer screening cases in 2021 involve the total number, with colon cancer, oral cancer, cervical cancer and breast cancer all having decreased significantly when compared with 2020 during same period, as total cancer screening numbers decreased from 307,547 to 103,289 (66% decrease). There was a 63%, 79%, 65% and 71% decrease in screening cases of colon cancer, oral cancer, cervical cancer and breast cancer, respectively, during this period. We also examined the change in cancer screening cases between 2019 to 2021 to confirm the trend, which again revealed a significant decrease. Total cancer screening numbers decreased 70% (2019–2021), with decreases of 65%, 83%, 70% and 76% in colon cancer, oral cancer, cervical cancer and breast cancer being seen, respectively. The largest decrease was noticed in oral cancer no matter which period we examined, including 2019 to 2021 and 2020 to 2021 (79% and 79%).

**Table 1:** Screening numbers in 2019, 2020 and 2021

	**May to July, 2019(n, %)**		**May to July, 2020(n, %)**		**May to July, 2021(n, %)**		Difference of 2019–2021(%)	Difference of 20**20**–2021(%)
**Total screening amount**	348,732		307,547		103,829		-70%	-66%
**Colon cancer**	172,197	(49.4%)	160,151	(52.1)	59,974	(57.8%)	-65%	-63%
**Oral cancer**	36,973	(10.6%)	28,841	(9.4)	6,139	(5.9%)	-83%	-79%
**Cervical cancer**	70,113	(20.1%)	61,608	(20.0)	21,345	(20.6%)	-70%	-65%
**Breast cancer**	69,449	(19.9%)	56,947	(18.5)	16,371	(15.8%)	-76%	-71%

There was a significant decrease in screening rates seen in the Taipei Divisions when compared with the other areas no matter which cancer was studied (Table 2). The proportion of screening cases regarding total, colon cancer, oral cancer, cervical cancer and breast cancer decreased much more in the Taipei Divisions when compared with other areas in 2020 to 2021 (74% vs 63%, 75% vs 57%, 84% vs 77%, 68% vs 64% and 73% vs 71%), and during 2019 to 2021 (76% vs 68%, 74% vs 62%, 89% vs 81%, 73% vs 68%, 80% vs 74%), both respectively. Cases of oral cancer screening decreased the most no matter which area or period we compared; in 2020 to 2021 (84% in the Taipei Divisions and 77% outside of Taipei) and 2019 to 2021 (89% in the Taipei Divisions and 81% outside of Taipei). A proportional decrease in screening cases was noticed in all areas when confirmed COVID-19 cases rose (Figs. 1 and 2). The slope is much steeper in the Taipei Divisions then the other divisions. A relatively higher R square was also noticed during 2019 to 2021.

**Table 2:** Screening numbers in regions and cancer types during 2019, 2020 and 2021

	**May to July, 2019(n, %)**	**May to July, 2020(n, %)**	**May to July, 2021(n, %)**	**Difference of 2019–2021(%)**	**Difference of 2020–2021(%)**
**Total amount in Taipei**	107,184	97,810	25,245	-76%	-74%
**Total amount out of Taipei**	235,766	204,017	76,167	-68%	-63%
**Colon ca. in Taipei**	50,684	54,056	13,293	-74%	-75%
**Oral ca. in Taipei**	10,155	7,308	1,144	-89%	-84%
**Cervical ca. in Taipei**	23,027	19,334	6,206	-73%	-68%
**Breast ca. in Taipei**	23,318	17,112	4,602	-80%	-73%
**Colon ca. out of Taipei**	118,837	104,962	44,763	-62%	-57%
**Or**al **ca. out of Taipei**	26,352	21,375	4,960	-81%	-77%
**Cervical ca. out of Taipei**	46,497	41,834	15,018	-68%	-64%
**Breast ca. out of Taipei**	44080	38,853	11,426	-74%	-71%

## Discussion

We found a significant decrease in the number of screening cases between May and July in 2021 no matter which year we compared that period to, as there was a rise in COVID-19 confirmed cases and a Level 3 alert was implemented in Taiwan, representing an advance in epidemic prevention measures. No matter how many cases of COVID-19 were confirmed inside a particular area, screening cases dropped significantly in all areas of Taiwan, particularly in Taipei where most COVID-19 cases in Taiwan were being found. A proportional decrease in screening numbers was noticed in all areas.

Unlike most other countries, Taiwan had a quick response to COVID-19 and implemented a strong restriction policy in order to fight the epidemic. While sporadic cases were noticed during 2019–2021, small numbers of outbreaks were still found. A Level 3 alert was applied in May 2021, so the postponing of unnecessary and non-emergency hospital visits was propagated. Therefore, a drop in screening numbers was expected and subsequently confirmed in our research. Screenings for colon cancer, oral cancer, cervical cancer and breast cancer were evaluated separately, with the largest rate drop being noticed in oral cancer screening. As patients were required to remove masks and open mouths when undergoing an oral examination, this practice may have allowed people to become exposed and be at a greater risk of COVID-19 infection. Previous research has demonstrated the impact of COVID-19 on the screening of other cancers, particularly breast cancer [15, 18–20] and colon cancer [15, 17]. In our research, a great reduction in screening numbers was also noticed for both cervical cancer and oral cancer (2020–2021 cervical cancer and oral cancer: a drop of 65% and 79%; 2019–2021 cervical cancer and oral cancer: a drop of 70% and 83%, each respectively). Screening numbers rebounded once COVID-19 cases had dropped gradually after July 2022. (Fig. 3) Additionally, different regional impacts were also noticed with a greater decreasing rate being seen in the Taipei Divisions. (Figs. 1 and 2) Screening numbers in other divisions also dropped when COVID-19 cumulative cases increased. This may imply that the impact of COVID-19 has been universal and extensive. This innovative finding is important and will have a far-reaching impact on cancer screening policies in the future.

We found there was a great reduction in screening numbers accompanied with a rise of COVID-19 cases in Taiwan. Similar to previous research results, a decline in screening numbers corresponded to different COVID-19 incidence rates across different regions [15]. This may imply that a rise in incidences of COVID-19 had a great impact on cancer screening numbers. However, public health policy should also be considered as a reason. As mentioned above, there was a strict epidemic prevention policy put in place in Taiwan, which may have reduced the will of its citizens to leave their homes and seek medical resources if they did not have an emergent need. While this may have also reduced the rate of screenings at same time, this effect may have been seen on a national level. As for screenings being performed in clinics and hospitals, there were several community screening activities held regularly prior to the era of the COVID-19 pandemic in Taiwan. These activities were however immediately later shut down due to the onset of the COVID-19 pandemic [20]. Thus, a great reduction in screening numbers was recorded everywhere in Taiwan, particularly in Taipei.

Previous research has demonstrated the effect of COVID-19 on breast cancer screening [21], and significant delays in early breast cancer detection was thus noticed. Screening for early breast cancer, including AJCC stage 0 and stage 1, decreased by approximately 51% and 27%, respectively, when compared with the previous year of 2020 in Taiwan, while its effect on other cancer screenings and regional differences was not well evaluated. Having discovered a large screening gap during 2021 in Taiwan, actions should be taken to call back suitable screening candidates for regular cancer screening. Additionally, the longer lasting effects of COVID-19 on other cancer screenings should also be analyzed in the future.

There were some strengths surrounding this study. First, we enrolled data regarding all cancer screenings, which was a concept promoted by the government in Taiwan. This may have helped us to differentiate whether the effect of COVID-19 on cancer screening was universal or not. Second, we had enrolled cancer screening data not only from 2021 and 2020, but also 2019, so the screening reduction rate was calculated not only between 2021 and 2020, but also between 2021 and 2019. Additionally, we also compared different screening conditions between the period of the pandemic and the previous non-pandemic period. This may have more strongly validated our research, with the possibility of normal fluctuation being alleviated. Third, subgroup data, including different regions and different types of cancer screenings were also analyzed, thus allowing for a complete picture regarding the impact COVID-19 had on cancer screening, possibly allowing it to become completely uncovered.

There were still some limitations however to this study. First, we only enrolled screening data taken from insured individuals in Taiwan. But since most citizens in Taiwan are covered by National Health Insurance, this may have only had a small effect. Second, this is a retrospective cross-sectional study, so causality should be interpreted carefully. Third, other demographic characteristics were not collected. Fourth, demographic data surrounding COVID-19 cases and cancer screening were not fully collected, as residence locales of COVID-19 and cancer screening patients were not obtained. It should be noted that screening willingness may be evaluated in future studies in order to confirm the actual reason(s) for decreased screening numbers. Fifth, a lack of data surrounding cancer survival rates and cancer stage distribution is also an additional limitation, as a complete relationship between a reduction in rates for cancer screening and mortality rates or cancer stages could not be evaluated. Sixth, it is hard to explain why the number of people undergoing cancer screenings increased during a decrease in confirmed cases of COVID-19 (as indicated in Figs. 1 and 2).

According to previous data, no obvious growth regarding the number of cancer screenings was noticed (refer to Fig. 3). The relative increase in the number of cancer screenings in 2021 may be the result of a small outbreak of COVID-19 in 2020. However, the increase can instead be considered a normal variation. Further investigation, over a longer period of time, may help to illuminate the matter. Finally, COVID-19 had a small outbreak in the year 2020, although the outbreak’s impact and difference were not fully discussed. More research still needs to be done in order to better illustrate the difference from the 2021 outbreak and its true impact.

## Conclusion

Cases regarding colon, oral, cervical and breast cancer screening all declined rapidly in May through June of 2021 throughout Taiwan nationally, but was seen to be much more prominent in Taipei where most COVID-19 cases were diagnosed in Taiwan. Incidence rates may have had a great influence on, and a proportional decline in, screening numbers. Screening numbers for oral cancer dropped the most amongst the four types of cancer analyzed. Any effects resulting from COVID-19 on long-term health still needs to be more carefully evaluated, with efforts also being taken to address the deficits being seen in screening numbers during the COVID-19 pandemic.

## Data Availability

The datasets used and analysed during the current study are available from the corresponding author on reasonable request.

## References

[1] WHO Coronavirus (COVID-19) Dashboard. World Health Organization; 2022. Available from: https://covid19.who.int/.

[2] Umakanthan S, Sahu P, Ranade AV, Bukelo MM, Rao JS, Abrahao-Machado LF, Dahal S, Kumar H, Kv D (2020). Origin, transmission, diagnosis and management of coronavirus disease 2019 (COVID-19). Postgrad Med J.

[3] Wang CJ, Ng CY, Brook RH (2020). Response to COVID-19 in Taiwan: big data analytics, new technology, and proactive testing. JAMA.

[4] Thornton J (2020). Covid-19: A&E visits in England fall by 25% in week after lockdown. BMJ.

[5] Ateev Mehrotra MEC, David Linetsky, Hilary Hatch, David A. Cutler. The impact of the COVID-19 pandemic on outpatient visits: a rebound emerges. Commonwealth Fund; 2020. Available from: https://www.commonwealthfund.org/publications/2020/apr/impact-covid-19-outpatient-visits.

[6] Melonie Heron PD. Division of Vital Statistics. National vital statistics system: mortality statistics: Centers for Disease Control and Prevention, National Center for Health Statistics; 2021. Available from: https://www.cdc.gov/nchs/data/nvsr/nvsr70/nvsr70-09-508.pdf.

[7] Siu AL (2016). Screening for breast cancer: U.S. Preventive services task force recommendation statement. Ann Intern Med.

[8] Curry SJ, Krist AH, Owens DK, Barry MJ, Caughey AB, Davidson KW (2018). Screening for cervical cancer: US preventive services task force recommendation statement. JAMA.

[9] Davidson KW, Barry MJ, Mangione CM, Cabana M, Caughey AB, Davis EM (2021). Screening for colorectal cancer: US preventive services task force recommendation statement. JAMA.

[10] Krist AH, Davidson KW, Mangione CM, Barry MJ, Cabana M, Caughey AB (2021). Screening for lung cancer: US preventive services task force recommendation statement. JAMA.

[11] Yen AM, Tsau HS, Fann JC, Chen SL, Chiu SY, Lee YC (2016). Population-based breast cancer screening with risk-based and universal mammography screening compared with clinical breast examination: a propensity score analysis of 1 429 890 Taiwanese Women. JAMA Oncol.

[12] Su SY, Huang JY, Ho CC, Liaw YP (2013). Evidence for cervical cancer mortality with screening program in Taiwan, 1981–2010: age-period-cohort model. BMC Public Health.

[13] Huang CC, Lin CN, Chung CH, Hwang JS, Tsai ST, Wang JD (2019). Cost-effectiveness analysis of the oral cancer screening program in Taiwan. Oral Oncol.

[14] Chen MK, Hung HF, Duffy S, Yen AM, Chen HH (2011). Cost-effectiveness analysis for Pap smear screening and human papillomavirus DNA testing and vaccination. J Eval Clin Pract.

[15] Chen RC, Haynes K, Du S, Barron J, Katz AJ (2021). Association of cancer screening deficit in the United States with the COVID-19 pandemic. JAMA Oncol.

[16] Maringe C, Spicer J, Morris M, Purushotham A, Nolte E, Sullivan R (2020). The impact of the COVID-19 pandemic on cancer deaths due to delays in diagnosis in England, UK: a national, population-based, modelling study. Lancet Oncol.

[17] Cheng SY, Chen CF, He HC, Chang LC, Hsu WF, Wu MS (2021). Impact of COVID-19 pandemic on fecal immunochemical test screening uptake and compliance to diagnostic colonoscopy. J Gastroenterol Hepatol.

[18] Tsai HY, Chang YL, Shen CT, Chung WS, Tsai HJ, Chen FM (2020). Effects of the COVID-19 pandemic on breast cancer screening in Taiwan. Breast.

[19] Peng SM, Yang KC, Chan WP, Wang YW, Lin LJ, Yen AM (2020). Impact of the COVID-19 pandemic on a population-based breast cancer screening program. Cancer.

[20] Hsiu-Hsi C (2015). Instruction for community integrated screening.

[21] Chou CP, Lin HS (2021). Delayed breast cancer detection in an Asian Country (Taiwan) with low COVID-19 incidence. Cancer Manag Res.

